# Pullulan/Poly(vinyl alcohol) Hydrogels Loaded with *Calendula officinalis* Extract: Design and In Vitro Evaluation for Wound Healing Applications

**DOI:** 10.3390/pharmaceutics15061674

**Published:** 2023-06-07

**Authors:** Irina Mihaela Pelin, Mihaela Silion, Irina Popescu, Cristina Mihaela Rîmbu, Gheorghe Fundueanu, Marieta Constantin

**Affiliations:** 1 “Petru Poni” Institute of Macromolecular Chemistry, Gr. Ghica Voda Alley 41A, 700487 Iasi, Romania; impelin@icmpp.ro (I.M.P.); silion.mihaela@icmpp.ro (M.S.); ipopescu@icmpp.ro (I.P.); ghefun@icmpp.ro (G.F.); 2 Faculty of Veterinary Medicine “Ion Ionescu de la Brad”, University of Life Science, 8 Mihail Sadoveanu Alley, 707027 Iasi, Romania; crimbu@yahoo.com

**Keywords:** pullulan/poly(vinyl alcohol) hydrogels, *Calendula officinalis* extract, antibacterial, antioxidant, drug delivery system

## Abstract

The therapeutic efficiency of plant extracts has been limited by their poor pharmaceutical availability. Hydrogels have promising potential to be applied as wound dressings due to their high capacity to absorb exudates and their enhanced performance in loading and releasing plant extracts. In this work, pullulan/poly (vinyl alcohol) (P/PVA) hydrogels were first prepared using an eco-friendly method based on both a covalent and physical cross-linking approach. Then, the hydrogels were loaded with the hydroalcoholic extract of *Calendula officinalis* by a simple post-loading immersion method. Different loading capacities were investigated in terms of the physico-chemical properties, chemical composition, mechanical properties, and water absorption. The hydrogels exhibited high loading efficiency due to the hydrogen bonding interactions between polymer and extract. The water retention capacity as well as the mechanical properties decreased with the increase in the extract amount in hydrogel. However, higher amounts of extract in the hydrogel improved the bioadhesiveness. The release of extract from hydrogels was controlled by the Fickian diffusion mechanism. Extract-loaded hydrogels expressed high antioxidant activity, reaching 70% DPPH radical scavenging after 15 min immersion in buffer solution at pH 5.5. Additionally, loaded hydrogels showed a high antibacterial activity against Gram-positive and Gram-negative bacteria and were non-cytotoxic against HDFa cells.

## 1. Introduction

Biocompatible, non-toxic, and non-allergenic hydrogels with controlled flexibility and porosity and good exudate absorbing ability have been extensively studied and recommended for wound dressing materials or for skin tissue engineering and regeneration [1,2,3,4].

Due to the abundance of natural resources that are readily available, cheap, and almost without side effects, greater attention was given to plant extracts that proved to be efficient in the treatment of infectious diseases [5]. Increasing attention is paid to herbal medicine because the phytochemical constituents exert antimicrobial or antifungal activity, act as antioxidants and as free radical scavengers, and can increase cell proliferation, angiogenesis, and the synthesis of DNA and collagen [6]. Taking into account the poor bioavailability of aqueous or hydroalcoholic plant extracts, their rapid-burst release or long-term volatility, designing a support that can immobilize and gradually release the extract at the wound site is a real challenge. In this respect, hydrogel formulations containing plant extract, such as *Salix alba* [7], *Nigella sativa* [8], *Cryphaea heteromalla* [9], *Lawsonia inermis* [10], *Moringa oleifera* [11], *Raphanus sativus* [12], *Rosmarinus officinalis*, *Achillea millefolium*, *Calendula officinalis* [13,14], or *lavender* extract [15], were investigated for topical applications against skin pathogens.

*Calendula officinalis,* known as pot marigold, has been traditionally used in the treatments of inflammations of internal organs, gastrointestinal ulcers, and dysmenorrhea; as a diuretic and diaphoretic in convulsions; for inflammations of the oral and pharyngeal mucosa; and for wounds and burns [16,17,18,19]. Its flower extracts were proved to have antioxidant and anti-inflammatory properties due to the content of carotenoids, terpenoids and terpenes, flavonoids, quinones, coumarins, and other constituents; moreover, it has been reported to have good antimicrobial properties [20]. The European Medicines Agency (EMA) has approved the use of a lipophilic and aqueous alcoholic extract of this flower for the treatment of minor inflammation of the skin and for minor wound healing [21]. Typically, it has been used as such as a topical formulation; however, to increase the pharmaceutical availability, new methods of incorporation and delivery have been developed, for example the incorporation into a polymer matrix, film, or composite [20,22,23,24,25,26,27,28]. Moreover, since the increase in microbial resistance to antibiotics is a major problem, materials containing active compounds with antibacterial effects can be advantageous alternatives.

Hydrogels, due to their hydrophilic nature, display high swelling capacity and retain a large amount of water, making them able to keep the wound bed moist and to favor skin re-epithelialization [29,30,31]. Moreover, the use of hydrogels as pharmaceutical devices/materials is advantageous as they can be stored in the lyophilized form and can be swollen and impregnated with plant extracts immediately before use. For wound treatment, many polysaccharide-based hydrogels, in addition to their high swelling capacity, possess very good biological properties such as biodegradability, biocompatibility, and non-toxicity [32]. Moreover, polysaccharides are found in various renewable natural sources and due to their excellent physico-chemical and biological properties are extensively explored in the fabrication of hydrogels for medical purposes [33,34,35].

Among them, pullulan is frequently used as polymeric support, because is a non-toxic, non-immunogenic, non-carcinogenic, and non-mutagenic polysaccharide, with a great potential in industrial applications, as well as the food, pharmaceutical, cosmetic, and biomedical fields [36]. The main drawback of hydrogels synthesized from this polymer is their weak mechanical properties. Therefore, pullulan is often combined with synthetic polymers such as poly(vinyl alcohol), which is a water-soluble long-chain polymer intensively used for obtaining hydrogels with low toxicity, high water absorption, high elastic modulus, high mechanical strength, and good biocompatibility [37].

The purpose of the present study was to formulate a new hydrogel containing *Calendula officinalis* extract, with appropriate swelling, flexibility, and adhesive properties, ensuring simultaneously an extended contact with the inflammatory wound and a prolonged release of the active biomolecules. Evaluations of the relevant physico-chemical characteristics, the in vitro biopharmaceutical properties of the released plant extract, as well as the scavenging activity and antibacterial properties along with the cytotoxic effect were performed (considering potential use in skin topical applications).

The novelty presented in the article consists in the use of *Calendula officinalis* hydroalcoholic extract as the active principle to be post loaded within polymeric hydrogels. Moreover, hydrogels were synthesized by an eco-friendly method that cumulated in excellent swelling, bioadhesive, and mechanical properties as well as antioxidant and antibacterial activity.

## 2. Materials and Methods

### 2.1. Materials

The pharmaceutical *Calendula officinalis* L. (Asteraceae) flower extract (*C. officinalis*) (1:5 hydroalcoholic extract, ethanol:water = 52/48, *w*/*w*) was supplied by Dacia Plant S.R.L (Brasov, Romania). Pullulan (P) with Mw = 200,000 g/mol was purchased from Hayashibara Lab. Ltd. (Okoyama, Japan). Poly(vinyl alcohol) (PVA) with Mw = 47,000 g/mol and degree of hydrolysis 98.0–98.8 mol%, trisodium trimethaphosphate (STMP), and sodium hydroxide were purchased from Sigma–Aldrich Chemie GmbH, Steinheim, Germany. Folin–Ciocalteu’s phenol reagent (2N), 2,2-diphenyl-1-picrylhydrazyl (DPPH), gallic acid (GA), quercetin (Q), and oleanolic acid (OA) were obtained from Sigma Aldrich Co. (St. Louis, MO, USA), ascorbic acid was from Soharlau chemie (Barcelona, Spain). All chemicals were used as received without any further purification.

### 2.2. Preparation of Pristine P/PVA Hydrogels

The hydrogels were prepared following a method already published [38]. Briefly, 15% (*w*/*v*) PVA aqueous solution was prepared by dissolving 3 g polymer in 20 mL of distilled water at 90 °C under stirring. Then, 1 g of pullulan was added, so as to obtain a gravimetric ratio P/PVA of 25/75. After cooling the solution at room temperature, 1 mL of 10M NaOH and 5 mL of a 10% (*w*/*v*) STMP aqueous solution were added. After stirring for 10 min, the solutions were poured into 6 cm diameter Petri dishes and subjected to three cycles of freeze (18 h at −20 °C) and thaw (6 h at 25 °C). At the end, the hydrogels were intensively purified by washing with water over 48 h (changing the water four times a day), then cut into 1 cm diameter discs and finally recovered by lyophilization using a laboratory freeze dryer ALPHA 1-2 LD, Martin Christ, Germany.

### 2.3. Preparation of Calendula Extract—Loaded P/PVA Hydrogels

Stock hydroalcoholic solutions (ethanol:water = 50:50, *v*/*v*) as 5%, 10%, and 20% (*w*/*v*) of *C. officinalis* extract were prepared for the subsequent impregnation of the P/PVA hydrogels. Previously, the initial hydroalcoholic *C. officinalis* solution was evaporated to dryness using a rotary evaporator (30 °C and 30 bar), and the plant residue was dried for 24 h in a vacuum oven at 37 °C and then dissolved in appropriate volume of ethanol/water so as to obtain the desired concentrations. About 100 mg hydrogel sample (1 × 1 cm^2^) was immersed and maintained for 72 h in appropriate volumes of the stock *C. officinalis* extract solution so as to obtain a 1/1 gravimetric ratio between the two components. Then, the extract-loaded samples were kept in a ventilated hood for 12 h to ensure the evaporation of the alcohol and in dark to preserve the antioxidant capacity of the plant extract. Finally, the hydrogels were hydrated with distilled water, frozen at −20 °C, and then dried by lyophilization.

### 2.4. Characterization of Calendula officinalis Extract

#### 2.4.1. Identification of Phenolic and Triterpenic Compounds by HPLC-ESI MS

Separation and identification of phenolic compounds from *C. officinalis* extract was carried out by HPLC-ESI MS. The assays were performed with an Agilent 6500 Series Accurate-Mass Quadrupole Time-of-Flight (Q-TOF) LC/MS instrument, equipped with a binary pump, a UV-Vis detector DAD, and a Mediterranea Sea 18 column (4.6 mm × 150 mm, 5 μm particle size). A gradient of two solvents, (A) formic acid (0.1%, *v*/*v*) in water and (B) methanol, was used. Analysis conditions involving a linear gradient method for a total time of 90 min were used as follows: 0 min, 1% B; 2 min, 5% B; 10 min, 20% B; 40 min, 45% B; 55 min, 70% B; 75 min, 100% B; 80 min, 1% B; and then held for 10 min before returning to the initial conditions. The flow rate was 0.8 mL/min and 20 µL of samples was injected. The separation process was monitored by UV-VIS DAD detector at 280 nm, the eluate was then partitioned, and 0.1 mL/min was directed to ESI-Q-TOF MS.

Separation and identification of triterpenes in *C. officinalis* extract was conducted in a Phenomenex C18 Luna column (150 mm × 4.6 mm, 5 μm particle size) and monitored by UV-VIS DAD detector at 210, 254, and 360 nm. The mobile phase was composed of water (A) and formic acid (0.1%, *v*/*v*) in acetonitrile (B). Gradient elution of 10% B to 60% B in 47 min, then to 100% B in 60 min was conducted and then held for 10 min to return to the initial conditions. The flow rate was 1 mL/min, solvent split ratio 1:9 for ESI and 20 µL of samples was injected.

In both cases, the ESI-Q-TOF MS instrument was operated in positive and negative ion mode. The capillary voltage was set to 4 KV and source temperature to 325 °C. Nitrogen was used as nebulizer gas at 40 psi and as drying gas at a flow rate of 8 L/min. The full-scan mass spectra of the investigated compounds were acquired in the range *m*/*z* 50–1800 for phenolic compounds and *m*/*z* 100–3000 for triterpenes. Data were collected and processed using MassHunter Workstation Software Data Acquisition for 6200/6500 Series, version B.07.00 (Agilent Technologies, Inc., Santa Clara, CA, USA).

#### 2.4.2. Total Phenolic Content (TPC)

TPC of the *C. officinalis* extract was determined using the Folin–Ciocalteu protocol [39], slightly modified by the authors. First, a 1 mg/mL *C. officinalis* solution was prepared in a mixture of ethanol/water = 50/50 (*v*/*v*), then 1 mL from this solution was diluted with 9 mL distilled water. Finally, 0.5 mL Folin–Ciocalteu’s reagent, 1.5 mL 20% (*w*/*v*) Na_2_CO_3_, and distilled water until solution reached 25 mL were added successively in a volumetric flask. The solution was incubated in the dark for two hours at room temperature and the absorbance was measured (in triplicate) at 760 nm using a UV–Vis spectrophotometer (Specord 200 Analytik Jena, Jena, Germany).

TPC was expressed as mg gallic acid equivalents (GAE) per gram of dry *C. officinalis* extract, based on the calibration curve of freshly prepared gallic acid solution in the concentration range 5 ÷ 100 µg/mL (y = 0.0209x, R^2^ = 0.9991).

#### 2.4.3. Total Flavonoid Content (TFC)

TFC of the *C. officinalis* extract was determined considering the formation of the flavonoid complex between the flavonoid and aluminum chloride interaction, following a method previously described [40]. Therefore, 1 mL of 1 mg/mL *C. officinalis* hydroalcoholic solution was diluted with 5 mL distilled water, then 0.3 mL of 5% (*w*/*v*) NaNO_2_ solution was added under stirring. After 5 min, 0.6 mL of 10% (*w*/*v*) AlCl_3_ was added and the flask was kept for another 6 min in the dark. Finally, 2 mL of aqueous 1M NaOH was added and the mixture was diluted with water to a final volume of 10 mL. The flask was kept again in the dark for 10 min (until the color changed from yellow to pale pink).

The absorbance was measured at 510 nm and the TFC was expressed as mg quercetin equivalents (QE)/g of dry *C. officinalis* extract using a calibration curve of freshly prepared quercetin solution (y = 0.001x, R^2^ = 0.995).

#### 2.4.4. Total Triterpenes Content (TTPC)

The content of triterpenes in the *C. officinalis* extract was determined by the spectrometric method as reported in the literature [41]. Briefly, a volume of 50 µL of *C. officinalis* extract solution (10 mg/mL in ethanol/water = 50:50, *v*/*v*) was transferred into a capped test tube which was kept in a water bath heated at 85 °C until dryness. Then, 250 µL of vanillin solution in acetic acid 99.7% (50 mg/mL) and 500 μL of sulfuric acid (99.5%) were added in this order. The tube was immersed in a water bath at 60 °C for 30 min and then transferred into an ice bath followed by the addition of 2500 μL acetic acid (99.7%). The tube was maintained on ice for 20 min and then kept at room temperature for an additional 20 min. The absorbance was read at 540 nm using a UV–Vis spectrophotometer (Specord 200 Analytik Jena, Jena, Germany) equipped with quartz cells. The content of total triterpenes was expressed as mg equivalents of oleanolic acid (OA)/g of dry extract and determined using a standard calibration curve of oleanolic acid freshly prepared solution in ethanol (100 µg/mL) (y = 0.0262x, R^2^ = 0.9997).

### 2.5. Characterization of Unloaded and Calendula-Extract-Loaded P/PVA Hydrogels

#### 2.5.1. Chemical Composition

Pullulan content of the hydrogels was determined by phenol-sulfuric acid assay [42] using glucose and pullulan for the calibration curve. Firstly, 25 mg of P/PVA hydrogels was refluxed in 50 mL of 0.5M H_2_SO_4_ at 100 °C for 17 h. Then, in a test tube over 2 mL of this solution, 1 mL of phenol 5% and 5 mL H_2_SO_4_ 96% were added, thoroughly mixed, and left for 10 min until the brown color appeared. After cooling at room temperature in an ice bath, the solution absorbance was read at 490 nm and the content of pullulan in the sample was calculated based on a calibration curve.

The chemical composition of the hydrogels with or without *C. officinalis* extract was confirmed by FT-IR spectra recorded in the 4000–400 cm^−1^ range, using a FTIR Vertex 70 spectrometer (Bruker, Vienna, Austria). Lyophilized samples of hydrogels, unloaded or loaded with extracts, were mixed with KBr and pressed into pellets.

#### 2.5.2. Morphology and Porosity

The morphology and the mean size of the pores of the hydrogels with or without *C. officinalis* extract were investigated using a Quanta 200 Scanning Electron Microscope (FEI Company, Hillsboro, OR, USA). Images of the lyophilized hydrogels were obtained in the Secondary Electron Imaging (SEI) mode using a voltage of 20 kV.

The porosity of the hydrogels was determined by the liquid displacement technique according to Moshayedi et al. [43]. Briefly, the freeze-dried samples with known dry weights (*W*_0_, g) and volumes (V, cm^3^) were immersed in anhydrous ethanol at room temperature. After 5 min, the hydrogels were taken out from the medium and gently tapped with blotting paper for removal of the surface ethanol. Then, the porosity of the hydrogels was calculated using Equation (1):

(1) $$P\;(\%)=\frac{\left(W_{s}-W_{0}\right)}{\rho V}\times 100$$

where *W*_s_ represents the saturated weight of the hydrogels (g) and ρ denotes the density of anhydrous ethanol at room temperature (0.785 g/cm^3^).

#### 2.5.3. Swelling Ratio

The capacity of the hydrogels to swell in simulated skin condition media was evaluated from the swelling ratio as follows: 35 mg of dried hydrogels was immersed in phosphate buffer (PB, 0.0667 M KH_2_PO_4_ + Na_2_HPO_4_) with pH 5.5 at 32 °C. At different time intervals, the samples were taken out from PB solution and the excess of the aqueous media was removed with blotting paper. Finally, the samples were weighed and the swelling ratio (*SR*) and equilibrium swelling ratio (*SR*_eq_) were calculated following Equations (2) and (3):

(2) $$SR\;(\%)=\frac{W_{s}-W_{d}}{W_{d}}\times 100$$

(3) $${SR}_{eq}\;(\%)=\frac{W_{e}-W_{d}}{W_{d}}\times 100$$

where *W*_s_—weight of hydrogels in swollen form at time *t*; *W*_e_—weight of hydrogels in swollen form at equilibrium; and *W*_d_—weight of hydrogels in dried form.

#### 2.5.4. Mechanical Properties and Bioadhesiveness

*Compressive* test of the hydrogels with or without *C. officinalis* extract was performed using a Brookfield Texture PRO CT3(R) Analyser (Brookfield Engineering Laboratories Inc., Middleboro, MA, USA). Disc hydrogels, 15 mm in diameter and 7 mm height, were saturated with PB of pH 5.5 for two hours, then placed between two parallel plates and tested under uniaxial compression until 40% deformation, using 4500 g load cell and 0.5 mm/s speed test. The compressive strength was measured at 40% strain level, while the elastic modulus (E) was calculated from the linear part of stress–strain curves, between 5 and 10% of compression, using Equation (4):

(4) E=σε=FA∆ll0

where *σ*—compressive stress; *ε*—strain; *F*—force (N); *A*—cross-sectional area of the hydrogel (m^2^); Δ*l*—change in length; and *l*_0_—original length.

*Mucoadhesive* property was evaluated using a Brookfield Texture PRO CT3(R) texture analyzer. A chicken skin mucosa was used as the model surface, and PB of pH 5.5 at 32 °C was used as moistening fluid. The device consists of a tissue holder, where the mucosal tissue can be fixed between two plates. The upper plate has a 14 mm diameter hole in the center so that the probe with a 10 mm diameter can come into contact with the tissue. The test rig was placed in a beaker with buffer solution, and the desired temperature could be reached by using an electric plate. The hydrogel was attached to the lower side of the probe with double-sided adhesive tape. To start the experiment, the probe was lowered to the mucosa with a defined pretest speed. After reaching the trigger force, the probe with the hydrogel was pressed down to the mucosa at 0.5 mm/s, held for 60 sec with 1 N force, and the maximum force (F_max_) required for detaching the hydrogels was measured at the same speed (0.5 mm/s).

### 2.6. Loading Capacity and Calendula Extract Release from Hydrogels

The amount of *C. officinalis* extract incorporated in hydrogel was determined both gravimetrically and spectrophotometrically (λ = 325 nm, maximum absorption corresponding to flavones and flavonols present in the plant extract [44]) as the difference between the amount of extract initially added and that found in the dried hydrogel or the washing solvent after loading procedure [45]. The extract concentration was calculated using a previously constructed calibration curve of *C. officinalis* in ethanol/water (50/50, *v*/*v*) (y = 0.0029x − 0.0134, R^2^ = 0.9997) and the quantity of loaded *Calendula* in the hydrogel (loading capacity, LC) was evaluated by Equation (5):

(5) $$LC\;\left(\%\right)=\frac{Amount\;of\;extract\;in\;hydrogel}{Amount\;of\;extract-loaded\;hydrogel}\times 100$$

Additionally, the loading capacity of hydrogels was determined by quantifying the polyphenolic compounds contained in the *C. officinalis*-loaded hydrogels using the Folin–Ciocalteu test [39]. The hydrogels were weighed (~25 mg) and placed in 4 mL of hydroalcoholic solution (50/50, *v*/*v*) for 6 h in dark under gentle stirring (50 rpm). Then, the supernatant was replaced by another 2 mL each 8 h. After 72 h, 0.2 mL from the collected solution containing *C. officinalis* extract (20 mL) was assessed for TPC content following the protocol described for the free extract in Section 2.4.2.

The in vitro release study was carried out in PB of pH 5.5 at 32 °C, simulating skin conditions, for 24 h. The extract-loaded hydrogels (~25 mg) were immersed in 30 mL of the release medium, under gentle stirring (150 rpm). At different time periods, about 3 mL of release solution was removed and replaced with the same volume of fresh buffer. The concentration of *C. officinalis* extract released was determined by UV-Vis spectrophotometry at λ = 325 nm based on the standard calibration curve obtained under the same conditions (y = 0.0027x + 0.0045, R^2^ = 0.999).

The Korsmeyer–Peppas model described by Equation (6) [46] was used to analyze the release mechanism from various loaded hydrogels.

(6) $$\frac{M_{t}}{M_{inf}}=kt^{n}$$

where *M*_t_ is the cumulative amount of the *C. officinalis* extract released at time *t*, *M*_inf_ is the initial concentration of the extract in the P/PVA hydrogels, *k* is the Korsmeyer release constant dependent on the polymer characteristics, and *n* is an exponent which defines the mechanism of release, whether it is diffusion-controlled or both diffusion- and erosion-controlled. The value (*n*) was determined from the slope of the logarithmic plot of release rate versus logarithm of time (*t*).

### 2.7. Antioxidant Activity

The antioxidant activity of the *C. officinalis*-loaded hydrogels and pure extract was evaluated using the 2,2-diphenyl-1-picryl-hydrazyl (DPPH) method according to the literature [47,48,49].

First, 12 different concentrations from 2.5 to 300 µg/mL of *C. officinalis* were prepared from a mother solution of 1 mg/mL extract (ethanol/water = 50/50, *v*/*v*). Then, 0.5 mL of DPPH, freshly prepared in ethanol (0.004%), was added in each Eppendorf tube containing 1 mL of extract, shaken gently for 30 min in dark, and the absorbance was measured at 523 nm. Depending on the concentrations of *C. officinalis*, the color changed from purple to pale yellow, and the ability to scavenge the DPPH radical was calculated using Equation (7):

(7) $$\left(\%\right)\;inhibition=\frac{\left(A_{0}-A_{1}\right)}{A_{0}}\times 100$$

where *A*_0_ is the absorbance of the control (DPPH solution without extract), and *A*_1_ is the absorbance of extract solution with DPPH [48]. The analysis was repeated in triplicate and the IC_50_ values (concentration of the *C. officinalis* that causes 50% scavenging) were determined from the graph of inhibition percentage versus *C. officinalis* concentration.

The extent of antioxidant capacity of *C. officinalis*-loaded hydrogels [49] was measured for the hydrogel extract solution prepared by immersing the loaded hydrogels (5–10 mg) in 4 mL PB of pH 5.5 for different time periods in dark. Then, 1 mL of each extract hydrogel solution was mixed with 0.5 mL of ethanolic DPPH solution and the mixture was thoroughly mixed and kept in the dark for half an hour at ambient temperature. The analysis was repeated three times and the percentage of DPPH radical scavenging activity was calculated from Equation (7). Additionally, the standard method of assessing the DPPH scavenging activity was performed by immersing the extract-loaded samples (~6 mg) in 1.5 mL of ethanolic DPPH solution (0.004%) at room temperature in the dark. At specific time periods, the absorbance at 523 nm was read and the DPPH radical scavenging activity was calculated by using Equation (7).

### 2.8. Antibacterial Activity

Antimicrobial and antifungal activity of *C. officinalis*-loaded P/PVA hydrogels was evaluated by the Kirby–Bauer test and “time-kill” method.

For Kirby–Bauer disc diffusion method, the standardized bacterial cultures of *Staphylococcus aureus* ATCC 25923 (*S. aureus*), *Escherichia coli* ATCC 25922 (*E. coli*), *Pseudomonas aeruginosa* 9027 ATCC (*P. aeruginosa*), and *Candida albicans* ATCC 90028 (*C. albicans*) were prepared to a cell density corresponding to 0.5 McFarland turbidity standard (1.5 × 10^8^ CFU/mL). One milliliter of each microbial suspension was distributed into Petri dishes (90 mm), over which a Mueller–Hinton agar (Oxoid Ltd., Basingstoke, UK) culture medium was spread. Hydrogel discs (d = 5 mm, h = 2 mm) with or without *C. officinalis* extract, hydrated with 50 µL of sterile distilled water, were placed onto culture medium previously prepared. A standardized gentamicin disc (10 µg) was used as a positive control. All Petri dishes were incubated at 37 °C for 24 h and the antimicrobial activity was assessed by measuring the diameter of the inhibition zones formed around each sample.

The antibacterial activity of *C. officinalis* extract-loaded hydrogels was also assessed by “time-kill” assay adapted as follows: a total volume of 50 µL of bacterial suspension (7.5 × 10^4^ CFU) was added onto each dried hydrogel fragment (d = 5 mm, h = 2 mm). The samples were incubated in a thermostat at 37 °C. After 24 h, each hydrogel was immersed in one milliliter of nutrient broth, homogenized by vortexing, and incubated again at 37 °C for 24 h. After incubation, each milliliter of bullion was embedded in Mueller–Hinton agar culture medium, melted, and cooled to 45 °C. After solidification, the Petri dishes were incubated again at 37 °C. Then, (after 24 and 72 h) the plates were examined and the bacterial colonies (colony-forming units—CFU) that had survived after contact with the hydrogels containing different amounts of *C. officinalis* extract were counted. The antibacterial efficacy was determined by using Equations (8) and (9) [50]:

(8) $$Log\;reduction\;(LR)=mean\;Log\;\left(control\right)-mean\;Log\;(surviving\;population)$$

(9) $$\%\;Reduction=\frac{Control-Surviving\;population}{Control}\times 100$$

where *Control* represents the initial microbial population.

### 2.9. Cytotoxic Activity

Cytotoxic evaluation of hydrogels without and with *C. officinalis* extract was performed by MTT assay as follows: Human Dermal Fibroblasts (Thermo Fisher Scientific, Bleiswijk, The Netherlands) adult (HDFa) cells were cultured in Dulbeco’s modified Eagle medium (DMEM) supplemented with 10% FBS, 1% (*v*/*v*) streptomycin-penicillin, and 1% (*v*/*v*) non-essential amino acids, at 5% CO_2_ and 37 °C. The cells were allowed to 80% attach to the surface of 7 mL Nunc^TM^EasYFlask™ cell culture flasks (Thermo Fisher Scientific Co., Waltham, MA, USA) and then were trypsinized with 0.05% trypsin solution at 37 °C. After centrifugation and re-suspension in fresh medium, the viable cells were cultivated on 96-well plates, allowed to attach for 24 h prior to the treatment, and after that treated with various concentrations of the diluted extracts of hydrogel samples for 24 h. Thus, after the sterilization of the hydrogels by UV for 5 min, ~9 mg of unloaded or *C. officinalis*-loaded samples was incubated in 5 mL of culture medium for 24 h at 37 °C, after which the mixture was centrifuged for 5 min at 2000 rpm/min. Then, a series of dilutions equivalent to 100%, 50%, 33.3%, 16.6%, and 8.3% of the extract in the medium were added to the plate and incubated for 24 h. After the incubation time, 100 µL of culture medium was replaced with the same volume of fresh medium and 10 µL of MTT dye (from 5 mg/mL stock) was added to each well and incubated for 4 h at 37 °C with 5% CO_2_. Further, 90 µL of medium was removed and the formazan crystals, resulting from cellular reduction of MTT, were dissolved in DMSO (100 µL) solution and incubated for 10 min at 37 °C. The absorbance at 570 nm was measured using a Multiskan FC automatic plate reader (Biotek, Germany). Cell viability, expressed as % of untreated cells (control) considered 100% viable, was calculated according to Equation (10):

(10) $$Cell\;viability\;(\%)=\frac{Sample\;absorbance}{Control\;absorbance}\times 100$$

The average values of 3 independent experiments (each time all conditions were repeated with triplicate wells) were considered.

### 2.10. Statistical Analysis

The results are expressed as mean ± standard deviation (SD). Statistical differences between groups were analyzed using one-way ANOVA followed by *t*-test Tukey’s HSD (honestly significant difference) post hoc test.

## 3. Results and Discussion

### 3.1. Characterization of Calendula officinalis Extract

*C. officinalis* extract has a complex chemical composition, dominated by flavonoids and terpenoids. Triterpenes are mainly represented by oleanolic acid derivatives specific for this species: triterpenoid saponins, Calendulosides A–H, and triterpenoid glycosides [51].

The identification of phenolic compounds and triterpenes in *C. officinalis* extract was performed by HPLC-ESI MS using two different columns and procedures for their separation (Section 2.4.1). Initially, based on their specific retention time and mass spectra, sixteen phenolic compound standards (gallic acid, 3,4 DHB, chlorogenic acid, vanillic acid, caffeic acid, syringic acid, p-coumaric acid, cinnamic acid, cynarin, quercetin 7-rhamnoside, luteolin 7-O-glucoside, isoquercetin, apigenin 7-glucoside, kaempferol 7-O-glucoside, fisetin, and quercetin) were identified and analyzed using the chromatogram for mixed standards solution (ESI Figure S1).

**Figure 1:**
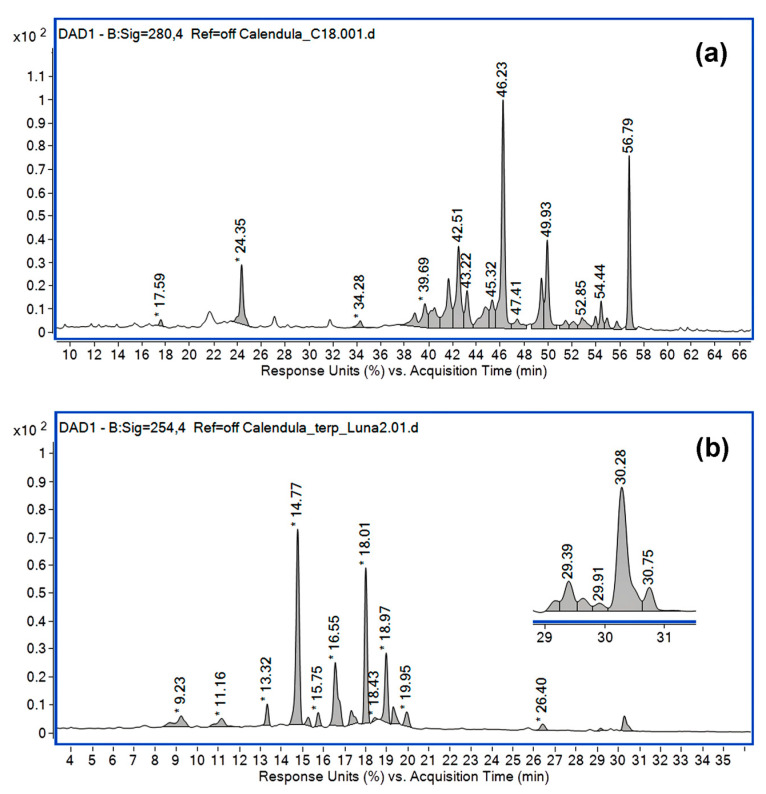
HPLC-DAD chromatograms for *Calendula officinalis* extract: (**a**) Mediterranea Sea 18 column, formic acid (0.1%, *v*/*v*) in water and methanol, 280 nm, ESI (+); (**b**) Phenomenex C18 Luna column, water and formic acid (0.1%, *v*/*v*) in acetonitrile, 254 nm, ESI (−).

The chromatographic separations of the phenolic compounds in *C. officinalis* extract, using a Mediterranea Sea 18 column, are depicted in Figure 1a. Positive electrospray ionization mode allowed the detection of many phenolic acids and flavonoid compounds. Chlorogenic acid, caffeic acid 3-glucoside, hydroxycaffeic acid, isorhamnetin-3-O-glucoside, isorhamnetin 3,7-di-O-β-D-glucopyranoside, and quercetin-3-O-galactoside were some of the most important compounds identified in this extract (ESI Figure S2).

Using a Phenomenex C18 Luna column and a separation method modified according to Budan et al. [52], it was possible to reveal the presence of some triterpenes in addition to a lot of flavonoids (also identified by the previous method) (Figure 1b).

Table 1 lists each identified compound based on its retention time and mass spectrum. The structure assignment of all compounds was based on a rigorous search for molecular ions using the extracted ion mass chromatograms and comparing those with the literature [52,53,54,55,56,57,58,59,60,61,62,63,64].

Olennikov et al., in their previous investigations, detected and isolated from *C. officinalis* leaves and flowers extracts a large part of the compounds found in this study, such as chlorogenic acid, isorhamnetin-3-O-glucoside, isorhamnetin 3,7-di-O-β-D-glucopyranoside, quercetin-3-O-galactoside, and typhaneoside [53,54,55]. Isorhamnetin-3-O-glucoside was detected for the first time in the flower extract of *C. officinalis* by Rigane et al. [56]. Typhaneoside, detected in our study at R_T_ = 42.5 min with [M + H]^+^ *m*/*z* 771.7, was previously identified by Cordova et al. [57] by comparison with an authentic sample previously determined in their laboratories. Andersen et al. also reported the presence of this flavonoid in *Calendula* extracts [58].

Besides phenolic compounds, three triterpenes were identified in the *C. officinalis* extract: oleanolic acid glucuronide D (*m*/*z* 793), oleanolic acid glucuronide C (*m*/*z* 955), and oleanolic acid glucuronide A (*m*/*z* 1117). The representative mass spectrum for triterpenes, recorded in the interval of 25–35 min, is shown in ESI Figure S3. All triterpenic compounds identified in this study are consistent with the data obtained by Budan et al. [52] and Mroczek et al. [63].

Many studies have shown that the content of polyphenolic compounds in plants is influenced by the genotype, soil conditions, and the difference in plant maturity [65]. Additionally, phenylpropanoid metabolism is affected by environmental conditions such as altitude, light, temperature, and soil forage content [66]. Consequently, the phytochemical evaluation of the *C. officinalis* extract of trade origin was performed by standard spectrophotometric methods. Based on the experimental results shown in Table 2, the content of total flavonoids in the investigated plant extract was higher (79.42 mg QE/g dry extract) than the content of phenols. The *C. officinalis* extract presented a phenolic content of 15.03 mg GAE/g of dry weight extract, close to that reported in the specialized literature [67]. Ćetković et al. determined a content of total phenols in the flower of *Calendula officinalis* L. (Serbia and Montenegro) of 15.12 mg/g of sample, and the content of total flavonoids (Markham) was 5.13 mg/g of sample [68]. On the other hand, Velicković et al. [47] determined a content of polyphenols in *Calendula officinalis* L. from the southeast region of Serbia that ranged from 29.79 mg of GAE/g of fresh flower petals extracted using ethanol/water 50/50, *v*/*v* (%), to 45.13 mg GAE/g in the water extract. The highest content of phenols was found in the aqueous extract. The flavonoid content is very close to that reported by Rigane et al. for *C. officinalis* flower extract from Tunisia, in methanol/water [56]. The content of triterpenoic compounds (53.58 mg OAE/g dry extract) was close to that reported by the EMA assessment report on *C. officinalis* L., flos. [21] and Budan et al. [52]. The results obtained using the spectrophotometric methods are in agreement with those found by HPLC-ESI MS analysis and highlight a relatively low phenol content in *Calendula* extract.

It has already been established that there is a strong relationship between total phenolic and flavonoid contents and the antioxidant activity of plant extracts [40,69]. Moreover, besides the anti-inflammatory action, terpenoid compounds are known for their antioxidant properties [51]. The lower the IC_50_ value of an antioxidant, the higher its free radical scavenging power. *C. officinalis* extract showed a strong antioxidant activity with values between 3.76 and 90.45% in the concentration range between 10 and 300 µg/mL, respectively (ESI Figure S4). An IC_50_ value of 122.67 µg/mL (Table 2) was determined, lower than that found for ascorbic acid (3.1 µg/mL) and quercetin (2.3 µg/mL), chosen as positive standards, and very close to that found by other authors [48,70].

### 3.2. Synthesis of P/PVA Hydrogels

As matrix for embedding the *C. officinalis* extract, a porous hydrogel based on P and PVA was prepared by a method previously described by Samoila et al. [38]. To obtain a support with both high swelling capacity and good mechanical properties, we synthesized a hydrogel combining a hydrophilic and biodegradable polysaccharide (P) with a synthetic hydrophilic polymer (PVA) at a weight ratio P/PVA of 25/75. Moreover, the hydrogel was synthesized by a double cross-linking procedure: covalent cross-linking with STMP and physical cross-linking by freeze–thawing cycles (Table 3). Unlike classic cross-linking agents, STMP is a safe and non-toxic cross-linker suitable for synthesizing polysaccharide-based hydrogels in alkaline medium [38,71]. On the other hand, freeze–thaw is a simple and harmless technique used to additionally stabilize the hydrogel and create pores in its structure [12].

The hydrogels were characterized by acid–base titration and spectrometric methods to assess their structural features. The chemical composition of H #0 hydrogel determined by the phenol-sulfuric acid [42] method showed a slight difference between the theoretical and practical content in P and PVA (Table 3). In fact, during the washing step, a fraction of un-cross-linked polymers (mainly the low molecular weight PVA) leaked from the hydrogel, as also proved by the gel fraction value. The presence of phosphate groups in the H #0 hydrogel confirmed the successful covalent cross-linking of the two polymers.

### 3.3. Loading of P/PVA Hydrogels

The entrapment of *C. officinalis* extract in P/PVA hydrogels was performed by a post-loading procedure. This method was preferred to the in situ loading of the polymer matrix to avoid the decomposition and leaching of plant extract during hydrogel preparation and purification.

The measure of loading amount is an essential parameter that is correlated with antimicrobial and antioxidant activity. The loading capacity of the prepared hydrogels with *C. officinalis* flower extract was examined both gravimetrically and by UV-Vis spectrometry and represented as the mean between the two values (Table 4). Moreover, the content of polyphenolic and triterpenic compounds in the collected samples was determined. The analysis of the results shows that the concentration of *C. officinalis* extract solution has a significant impact on the effectiveness of the loading process (Table 4). Thus, the addition of a more concentrated extract solution to the hydrogels increased the incorporation efficiency of the active substances. The largest amount of *C. officinalis* extract (737 mg/g) was entrapped by using a 20% (*w*/*v*) hydroalcoholic solution. The chemical affinity of the matrix components for the different *C. officinalis* extract compounds could be driven by their water solubility and intermolecular interaction with the functional moieties of the polymers in the hydrogel (hydrogen bonding interactions with OH groups) [72], but also by the “π-π” stacking among the plant phenolic compounds.

#### 3.3.1. FT-IR Spectra

**Figure 2:**
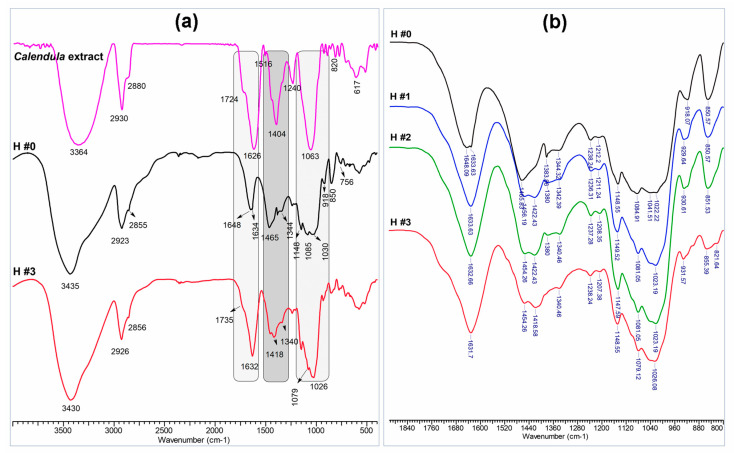
(**a**) FTIR spectra of *Calendula officinalis* extract, P/PVA hydrogel (H #0 sample), and *Calendula officinalis* 20% (wt.)-loaded P/PVA hydrogel (H #3 sample); (**b**) FTIR spectra region between 800 and 1900 cm^−1^ for the unloaded and *Calendula officinalis*-loaded P/PVA hydrogels.

In Figure 2a the comparative IR spectra of the *C. officinalis* extract, P/PVA hydrogel (H #0), and 20% *C. officinalis*-loaded P/PVA hydrogel (H #3) are depicted. The FTIR spectrum of *Calendula* extract presents a strong band at 3364 cm^−1^ assigned to the stretching of hydroxyl group (O–H), H-bonded stretching, which is characteristic of polyphenolic compounds [73]. The C-H asymmetric and symmetric stretching vibrations are observed at 2930 cm^−1^ and 2880 cm^−1^. The characteristic bands at 1724, 1626, 1516 cm^−1^, and 1446 cm^−1^ refer to the stretching vibration of C=O, the aromatic and olefinic C=C-C stretching modes, and indicate the presence of aromatic compounds in the extract. The band at 1412 cm^−1^ refers to the bending of C-H. In addition, the phenolic C–O stretching, typical of flavonoid C-rings, was observed at ~1240 cm^−1^ [74]. The bands at 1344 and 1404 cm^−1^ can be attributed, respectively, to the C-O-H angular deformations of phenols, a characteristic group of flavonoids [74], and to the C-O stretching in alcohol and ether bonds of monosaccharide units in the branched regions, such as galactose and glucan units, andthat the band at 1063 cm^−1^ can be attributed to the C-H group of carbohydrate monomers [24]. Finally, the out-of-plane bending bands of C-H in aromatic hydrocarbon were evident at 933, 820, 679, and 617 cm^−1^ [75]. These observations were also previously confirmed by Al-Mussawi et al. [76] and El-Hashemy et al. [77].

The IR spectrum of the pristine P/PVA hydrogel (H #0 sample) showed the presence of the characteristic absorption bands of both P and PVA components: a band in the region between 3200 and 3600 cm^−1^ (stretching vibration of the free O-H groups, intermolecular and intramolecular H bonds), 2923 and 2855 cm^−1^ (symmetrical and asymmetrical stretching C-H from CH_2_ and CH bonds), 1085 cm^−1^ and 1344 cm^−1^ (C-O stretching in PVA) [78], 1465 cm^−1^ (OH in-plane bending in pullulan skeleton), 1636 cm^−1^ (C–O–C bonds and glycosidic bridge) [79], and 1148 cm^−1^ and 1020 cm^−1^ (C-O-C stretching bonds in pullulan skeleton). The band observed at 1640 cm^−1^ is attributed to the presence of water in the samples. The band related to phosphate stretching (P-O-C) (1012 cm^−1^) is probably included in the 900–1140 cm^−1^ region overlapping those specific to pullulan [38,80]. The band around 918 cm^−1^ describes α-(1-6) and α-(1-4) linkages. The peak around 850 cm^−1^ indicates the α-configuration [81] and the absorption bands around 850 and 756 cm^−1^ show the pullulan’s 4C_1_ chair conformation.

The bands observed for *C. officinalis*-loaded P/PVA hydrogels (H #3 sample) were almost superimposable to those of pristine P/PVA (H #0 sample). However, the downshift in the −OH groups stretching from 3435 to 3430 cm^−1^, along with the intensification and the shift of C-O stretching both in PVA and P from 1085 to 1079 cm^−1^ and 1022 to 1026 cm^−1^, was observed. The peak at 1735 cm^−1^, attributable to an ester C=O stretching vibration, appears more intense as the *C. officinalis* extract concentration increases (Figure 2b). Additionally, the O-H bending of the phenols band at 1418 cm^−1^ appeared broadened and its intensity increased with the increase in the amount of plant extract loaded in the hydrogel. All these observations constitute proof of the plant extract’s presence in hydrogels, suggesting that its components were associated through H bonds involved in the OH of phenols and C=O of the C-ring [72,75,77].

#### 3.3.2. Morphology and Pore Size

The morphological characteristics of hydrogels (i.e., pore structure, pore size, and porosity) can affect their properties such as absorption capacity of water, swelling rate, mechanical strength, etc.

As already mentioned, chemically and mechanically stable P/PVA hydrogels were obtained following a double cross-linking method. The hydrogels, prepared in the absence of toxic organic solvents or chemical cross-linkers, have a homogenous internal network stabilized through chemical and physical interactions (hydrogen bonds).

**Figure 3:**
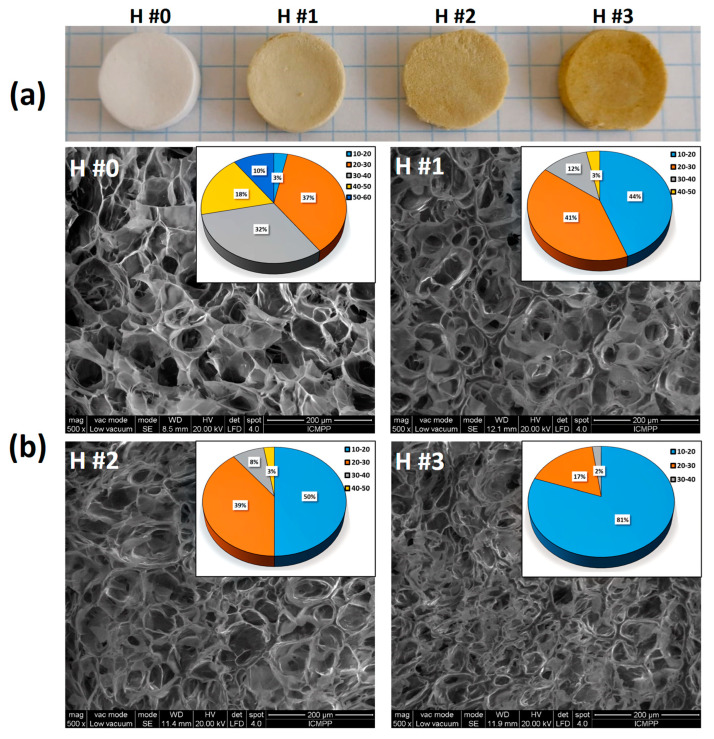
Optical (**a**) and SEM images (**b**) of cross-sections through the pristine P/PVA hydrogel (H #0) and the *Calendula officinalis*-extract-loaded P/PVA hydrogels (H #1, H #2, H #3). Insets in Figure 3b display pore size distribution diagrams.

The entrapment of *C. officinalis* extract in the P/PVA matrix was proved by analyzing the optical images of the loaded hydrogels (Figure 3a). With the increase in *C. officinalis* extract content, the color of the samples changed from white (H #0) to brown (H #3) with a progressive increase in color intensity.

The SEM image of the lyophilized hydrogel without *C. officinalis* extract (Figure 3b) shows a porous structure with interconnected cavity-like pores with irregular edges, with sizes varying between 10 and 60 μm with a mean pore diameter of 34.57 ± 10.34 µm (ImageJ Software version 1.53e). The walls of the cavity-like pores are not smooth or equal in dimension and express fibrous ends. By increasing the content of the extract within P/PVA hydrogels, the walls of the pores are less observable, with the pores being discontinuously covered with irregular islands of extract solution. The mean pore diameter decreased from 27.4 ± 6.98 (H #1) to 21.11 ± 6.30 (H #2) and 16.53 ± 4.97 µm (H #3). The size distribution diagrams (Figure 3b, insets) show a continuous narrowing of the size distribution with a notable increase in the fraction ranging between 10 and 20 µm (from 44 to 81%). The wall thickness was around 7.6 µm for all samples, regardless of whether they were loaded with extract or not.

The changes in porosity value for all samples are in accord with the SEM data. Hence, by entrapment of the *C. officinalis* extract, the porosity decreases from 42.89 ± 2.7% for H #0 to 37.25 ± 1.16% for H #1, 34.17 ± 2.64% for H #2, and 28.98 ± 1.97% for H #3.

#### 3.3.3. Swelling Behavior

**Figure 4:**
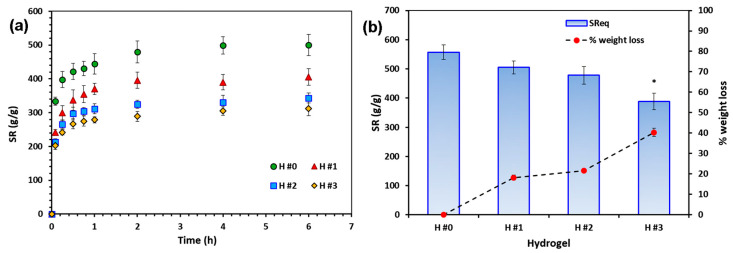
Swelling kinetics (**a**) and swelling ratio and weight loss at equilibrium (24 h) (**b**) of unloaded and *Calendula officinalis*-loaded P/PVA hydrogels. The measurements were performed in PB solutions with pH 5.5 at 32 °C and each point represents the average of the results from three different samples (*n* = 3). The * sign indicates a significant difference between groups (*p* < 0.05).

The swelling behavior of the P/PVA hydrogels was evaluated at pH 5.5 and 32 °C (Figure 4a), simulating skin physiological conditions. In this environment, we expected to have a dissociation of hydrogen bonds followed by polymer chain relaxation. As predicted, the *SR* values for all hydrogel samples increased rapidly in the first 2 h, the highest value being found for the H #0 hydrogels (without extract). The *C. officinalis*-loaded P/PVA hydrogels swelled less than H #0 sample due to the reduced mobility of polymer chains. In fact, the hydroxyl groups available to interact with water molecules were reduced, being involved in hydrogen bonds between polymers and *C. officinalis* extract (as demonstrated by FTIR spectroscopy shifts of the peaks assigned to -OH stretching vibrations (Figure 2)). The *SR* values after 24 h of incubation were 557%, 505%, 478%, and 388% for H #0, H #1, H #2, and H #3, respectively. A similar swelling behavior was previously reported by other researchers for PVA [82] or CS/PVA-based hydrogels [83] and films [49] loaded with plant extracts. They also stated that a decrease in *SR* values was due to the strong interactions between reactive functional groups in the polymeric matrix and extract.

It can be noticed that after 24 h, there were no significant differences between the *SR* values for H #0, H #1, and H #2 samples, indicating the release over time of some extract components from the hydrogels during the swelling test. These results were sustained by the weak yellow coloration of the PB solution during the test and some weight loss at the end of the experiment. Practically, after 24 h, sample H #1 reached approximately the same *SR* value as H #0, revealing that most of the extract was released in the swelling medium (Figure 4b). A similar phenomenon was previously reported for PVA hydrogels prepared by the freeze–thawing method and loaded with *Piper crocatum* extract [82].

#### 3.3.4. Mechanical Properties and Bioadhesiveness

The mechanical properties, along with an appropriate elasticity of hydrogels, are key factors in their potential application as dressing materials. Ideally, such healing materials must be flexible and durable and maintain their position while covering the wound. The adequate adhesion to the wound is enabled by materials with high elasticity, as this may affect the regeneration process [84].

**Figure 5:**
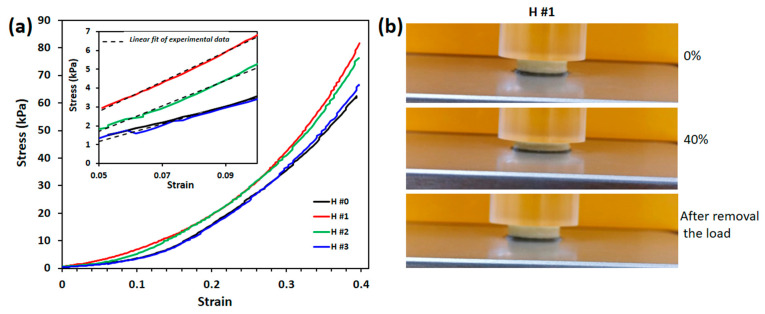
Compressive properties of unloaded and *Calendula officinalis*-loaded P/PVA hydrogels in the swollen state (**a**); the insets represent the linear dependence of stress−strain curves between 5 and 10% compressions, which was used to calculate the elastic modulus of all hydrogels. Photographs of H #1 hydrogel taken during the uniaxial compression test (**b**).

The effect of *C. officinalis* extract on the mechanical properties of P/PVA hydrogels is presented in Figure 5a. The elastic modulus of hydrogels was determined from the slope of linear dependence of stress−strain curves (inset of Figure 5a) and the data obtained are listed in Table 5. As can be observed, the elastic properties of the swollen hydrogels depend on the loading degree with *C. officinalis* extract. The compressive modulus of the pure P/PVA hydrogel (H #0) was lower (E = 42.53 KPa) than that of the *extract*-loaded hydrogels. The improved mechanical properties of the swollen hydrogels containing *Calendula* extract indicate the presence of hydrogen bonding between the functional groups (-OH) of the polymeric matrix and the extract, as already explained by FT-IR analysis and reported by other researchers [49,82,83]. The incorporation of 211.4 mg/g *C. officinalis* extract (H #1) causes the reduction in flexibility, and an increase in elastic modulus to 78.87 KPa and compressive strength to 81.55 KPa. However, the Young’s modulus of the hydrogels tended to decrease as the fraction of extract increased from 211.4 to 334.4 and 736.7 mg/g. The compressive strength (at 40%) of the hydrogel followed the same trend. In fact, a higher amount of *Calendula* components in hydrogel could hinder the interactions between the polymers and extract and create a denser and more inhomogeneous inner structure. Nevertheless, these results are in line with others found on in situ plant-extract-loaded hydrogels [82]. The hydrated hydrogels containing *C. officinalis* extract showed instant recovery after 40% compression and returned to the original shape, as shown in Figure 5b for H #1.

To evaluate the behavior of the *C. officinalis*-loaded P/PVA hydrogels in contact with the skin surface, bioadhesive tests were performed. It is well known that hydrogels can hydrate the skin and therefore can improve drug penetration [85]. Moreover, a bioadhesive hydrogel, due to its adhesion to the skin, will have a longer contact time at the site of application, improving the drug penetration and, consequently, the efficiency of the local therapy. The mucoadhesive performance of hydrogels was estimated from the maximal adhesive force (F_max_) [86] (Table 5). As can be observed, all hydrogels have beneficial adhesive properties; F_max_ values are in the required range for the adhesion process (0.3–1.3 N) [87]. However, the extract-loaded hydrogels were characterized by a significant increase (*p* < 0.05) in bioadhesiveness, the highest performance being observed for H #3 hydrogel. The incorporation of *C. officinalis* components leads to the increase in the hydrophobicity, but at the same time provides functional groups (-OH, -COOH, -NH_2_), facilitating mucoadhesion through hydrogen bonds or electrostatic interactions [88].

### 3.4. Release Kinetic of Calendula officinalis from P/PVA Hydrogels

**Figure 6:**
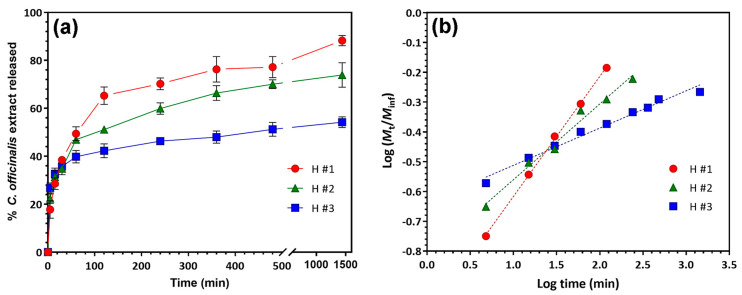
Release profiles of *Calendula officinalis* extract from P/PVA hydrogels in PB with pH 5.5 at 32 °C (**a**) and plots of Korsmeyer–Peppas model (**b**).

The release profiles of *Calendula officinalis* extract from hydrogels are shown in Figure 6a. A burst effect is observed in the first 15 min, when approx. 35% extract was released. The fast release can be caused by the rapid diffusion of the extract located on the surface of the P/PVA hydrogels. In the next 8 h, the release of *C. officinalis* extract takes place gradually but more slowly, the released percentage being under 80%: in particular, 77.22% from H #1, 70.07% from H #2, and 51.22% from H #3. Then, in the next 24 h, the extract is released very slowly, but still progressively, without noticing a tendency to re-bond on the polymer matrix. A significant difference between the release rates of the extract from the three hydrogels with different loading degrees must be emphasized. Consequently, the hydrogel with the highest loading degree showed the lowest release rate. After 24 h, H #3 released around 54.2% while H #2 and H #1 released 73.9 and 88.2%, respectively.

The release rates can be related to the hydrophobicity and degree of swelling of the P/PVA hydrogels in the saline release medium with pH 5.5 (see Figure 4), as well as to the hydrogen bonds between the active components of the extract and the polymer network. In fact, higher extract content in the hydrogel sample results in a higher hydrophobicity, more hydrogen bonds, and a lower swelling degree. These three factors act cumulatively and significantly reduce the release rates of the extract (Figure 6a).

The release kinetics of *C. officinalis* extract were analyzed by plotting the log of cumulative release data versus log of time (Figure 6b). The “*n*” values for the Korsmeyer–Peppas model were 0.4 for the extract released from H #1, 0.25 for H #2, and 0.12 for H #3, all “*n*” values being smaller than 0.5. These results indicate a quasi-Fickian diffusion mechanism, which means that the extract was released in a sustained manner [46].

### 3.5. Antioxidant Activity

The free radical scavenging capacity of *C. officinalis*-loaded P/PVA hydrogels was investigated by using the DPPH assays. The DPPH radical, a free radical with a maximum absorption band at 515–528 nm, is frequently used for the evaluation of the antioxidant activity of different compounds [89]. Due to its content of polyphenols, carotenoids, terpenoids, and flavonoids, *C. officinalis* flower extract can reduce the DPPH radical to diphenylpicrylhydrazine (yellow compound) (as demonstrated in Table 2), and can help in wound healing [90,91].

**Figure 7:**
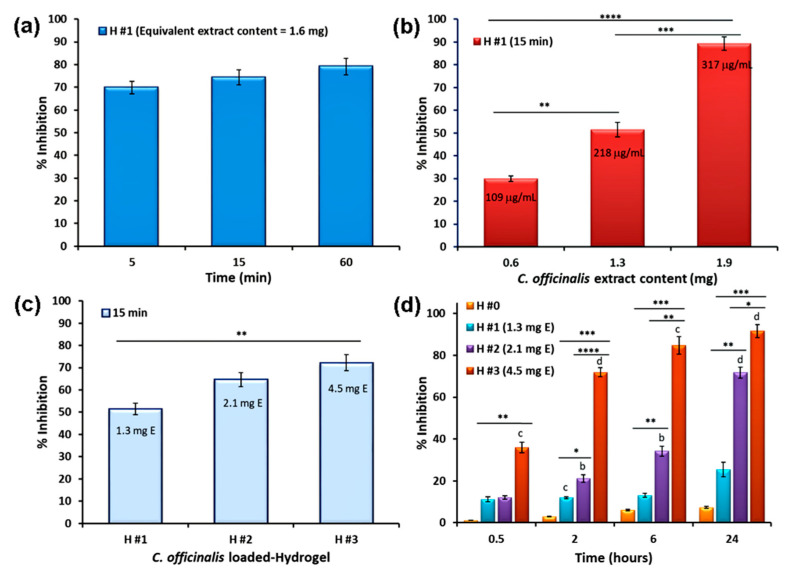
Antioxidant activity of P/PVA hydrogels containing *Calendula officinalis* extract as functions of (**a**) time immersion and (**b**) loading capacity, and (**c**) for different formulations. The experiments were performed in PB at 32 °C. Comparative radical scavenging activity for pristine P/PVA and *C. officinalis*-loaded P/PVA hydrogels by immersion in ethanolic DPPH solution (standard method) (**d**). Results are expressed as means ± standard deviation (S.D.) of three (*n* = 3) experiments. Values with different superscripts are significantly different vs. control (H #0); * *p* < 0.05, ** *p* < 0.01, *** *p* < 0.001, **** *p* < 0.0001 among samples.

Figure 7a–c show the DPPH radical scavenging activity of *C. officinalis* extract released from the hydrogels at different time periods of immersion in PB of pH 5.5 and 32 °C. Therefore, the DPPH free radical scavenging ability of the H #1 sample increased with the increase in time of immersion (Figure 7a). This behavior can be explained by the higher amount of active compounds released from the hydrogel into the buffer solution. In particular, the antioxidant activity after 15 min of immersion in PB solution was 74%, a value that is lower than that of free *C. officinalis* extract (90%) found at the same extract concentration (~300 µg/mL), but not significantly different (*p* < 0.05) to the value after 60 min of immersion (79%). By increasing the amount of H #1 sample (hence, *C. officinalis* content) (Figure 7b), a significantly stronger (*p* < 0.0001) free radical scavenging activity was observed. However, the inhibition percentage was almost 1.5 times lower for the released extract than that for the free extract, considering the same concentrations (ESI Figure S4). The effect of the content of extract in the hydrogel was also evidenced by comparing the scavenging activity of the three samples (Figure 7c): after 15 min of immersion, the radical inhibition activity increased from 52% for H #1 to 65% for H #2 and 72% for H #3. The increased free radical scavenging activity of the hydrogels resulted from a gradual increase in the released amount of the extract at this particular immersion time (Figure 6a).

Finally, we assessed the DPPH radical scavenging activity by the standard method [92] when the hydrogels came into contact, for different times, with the alcoholic DPPH solution. As shown in Figure 7d, the unloaded P/PVA hydrogels (H #0) presented a very weak antioxidant activity (7% after 24h), and this may be attributed to the intrinsic activity given by hydrogen donor –OH groups [93]. On the contrary, the antioxidant properties significantly increased with increasing amounts of *C. officinalis* extract in hydrogels. In fact, a larger quantity of extract means a higher amount of phenolic derivatives that enhance the hydrogen-donating ability—the basic phenomenon of the DPPH scavenging method [94]. Additionally, the scavenging activity increased with time and the maximum values were obtained after 24 h of incubation (26% for H #1 (phenol concentration 40 µg/mL), 72% for H #2 (phenol concentration 57 µg/mL), and 92% for H #3 (97 µg/mL phenol concentration)). Our results are in agreement with other results obtained on alginate/gelatin hydrogels loaded with guava leaf extract [95] and chitosan/PVA films loaded with *Ocimum tenuiflorum* [83]. For example, the composite hydrogels with a DPPH^•^ clearance ratio of about 55.4% [96] showed high antioxidant properties that scavenged ROS and RNS and, as showed by in vivo studies, sequentially eliminated inflammation, inhibited wound infection, and promoted wound healing. Since our hydrogels have a higher DPPH radical scavenging activity (74%), we expect to have at least the same properties.

### 3.6. Antibacterial Activity

The antibacterial activity of *C. officinalis*-loaded hydrogels was evaluated against the most common Gram-positive and Gram-negative bacteria species found in chronic wounds and also on a representative fungi, *Candida albicans* [97]. The zone diameters of susceptibility testing results were categorized as sensitive, intermediate, or resistant based on the CLSi breakpoint criteria [98].

The results indicated an intermediate antimicrobial and antifungal activity, against all tested microorganisms, for the hydrogels loaded with the highest amount of plant extract, as shown in Table 6 and Figure S5. These results, together with the lack of an antibacterial effect of the empty H #0 hydrogel, demonstrated that *C. officinalis* extract was responsible for the antibacterial and antifungal activity of the loaded hydrogels. However, a more pronounced antibacterial effect against *S. aureus* compared with *E. coli* was observed, and the lowest effect was obtained against *P. aeruginosa* when a clear inhibition zone appeared only for the highest amount of extract (H #3 sample; 10.5 mg extract). The results are in accordance with the reports of Efstratiou et al. [99] for the ethanolic extract of *C. officinalis* petals. In fact, the weak sensitivity to the action of the antimicrobial agents of the two Gram-negative bacteria is due to intrinsic resistance mechanisms (low permeability at the outer membrane level) [100]. The same authors [99] reported an inhibition zone of 19–28 mm for *S aureus* and 14–18 mm for *E. coli* when testing paper discs loaded with 4.5 mg of extract. As shown in Table 6, for a 3.9 mg extract content (H #2 sample) we obtained an inhibition zone of 12 mm for *S. aureus* and 11.6 mm for *E. coli*. It can be concluded that the results are consistent, taking into account that the release of *C. officinalis* extract from the hydrogel in the agar medium is retarded or hindered, compared with the rapid diffusion from the paper disc. The yeast *C. albicans* showed low sensitivity to the *C. officinalis*-loaded hydrogels, the higher antifungal activity being observed also for the hydrogel with a higher loading. The results are in accordance with the moderate antifungal activity reported for *C. officinalis* extract against this strain [56,99].

Anyway, even if the disc diffusion method is usually used for routine antimicrobial susceptibility testing and gives important results for a first approximation, it has been demonstrated that non-polar antimicrobial compounds do not diffuse well from the plant extracts in the aqueous agar matrix used in agar diffusion studies [101]. Therefore, when studying plant extracts by the diffusion technique, it must be considered that antimicrobial compounds have different polarities [101,102] and cannot develop their full capacity.

**Figure 8:**
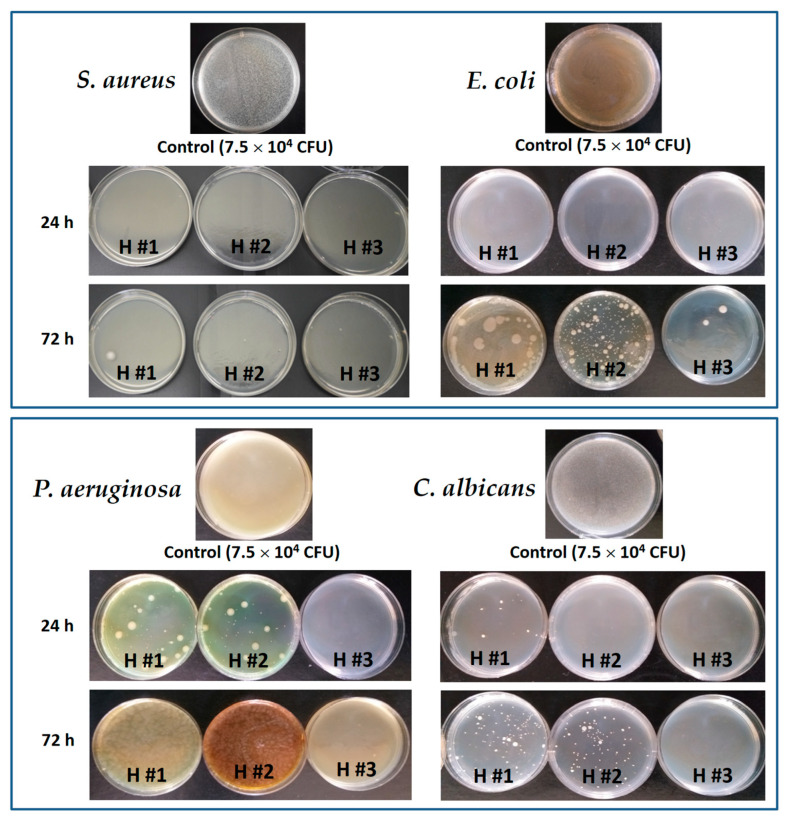
Antibacterial activity of P/PVA hydrogels containing *Calendula officinalis* extract after 24 and 72 h of incubation with *S. aureus*, *E. coli*, *P. aeruginosa*, and *C. albicans*.

In order to obtain information about the antibacterial activity of all plant extract compounds, a technique using a liquid medium was employed, where all samples were in contact with the bacterial suspension. The method consisted of the hydration of hydrogels loaded with *C. officinalis* extract with 50 µL of microbial suspension (7.5 × 10^4^ CFU/50 µL). Then, the antimicrobial activity of the hydrogels, measured by the total plate count method, was determined after 24 and 72 h of incubation in order to follow the bacteriostatic and bactericidal effects. The obtained results varied depending on the bacterial species and the concentration of the extracts in hydrogels, as presented in Figure 8 and Table 7.

As is well known, polyphenols and flavonoids can interact with the bacterial cell membrane through hydrophobic interactions, and so the lipid bacterial cell membrane becomes permeable and the leakage of cell content can take place [103]. It is also known that Gram-positive organisms are more susceptible than Gram-negative bacteria [103] and *C. officinalis* extract has a lower minimal bactericidal concentration against *S. aureus* (Gram-positive bacteria) compared with *E. coli* (Gram-negative bacteria) [56,104]. The P/PVA hydrogels loaded with *C. officinalis* extract showed a very good antibacterial activity against the suspension of *S. aureus*, inhibiting 100% of bacteria after 24 h of incubation. After 72 h, few colonies formed sporadically on the surface of the culture media, with the log reduction value also being very high (Table 7), demonstrating that hydrogels have bacteriostatic activity against this strain, regardless of the loaded amount of *C. officinalis* extract.

When testing the antimicrobial activity against *E. coli*, the microbial suspension was completely inhibited (100% reduction) after 24 h of contact with all hydrogels. This inhibitory (bacteriostatic) effect was not maintained up to 72 h (Figure 8), but the reduction percentage remained high: 99.33% for H #1 and even higher for the hydrogels with increased amounts of plant extract. The antibacterial activity against *P. aeruginosa* was lower compared with the other two bacterial strains, as also observed by the Kirby–Bauer diffusion tests. However, after 24 h of contact, the H #3 hydrogels inhibited microbial culture by 100% (bacteriostatic effect). The antimicrobial potential of extract-loaded hydrogels was also evident for H #1 (99.89%) and H #2 (99.94%). After 72 h of incubation, the microbial culture began to grow, but a log reduction of 2.4 (H #1) was maintained.

Due to the acquired resistance, *Candida albicans* is a yeast against which there are fewer and fewer effective antifungal agents [105]. The loaded hydrogels inhibited cell proliferation after 24 h, and the H #2 and H #3 samples were the most effective (reduction 100%); even the activity of H #1 (reduction 99.98%) is also significant. Moreover, the inhibitory activity of H #3 remained at 100% even after 72 h of incubation (possible microbicidal effect).

It turns out that the bacterial and fungal cells are inhibited as long as they are in contact with the antimicrobial compounds present in the hydrogels. However, the removal of the hydrogels from the site of action leads to the revitalization of the cells. Thus, the inhibition of cell proliferation for a prolonged period of time slows down the formation of new generations and limits the chance of microbial survival.

### 3.7. Cytotoxic Activity

**Figure 9:**
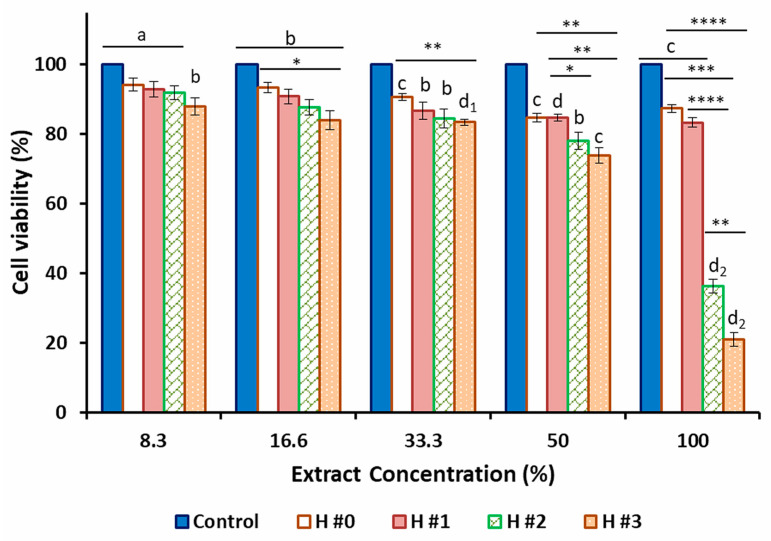
Viability of human dermal fibroblast adult (HDFa) cells exposed for 24 h to different concentrations of unloaded and *Calendula officinalis*-loaded P/PVA hydrogel extracts, assessed by MTT assay. Results are expressed as mean ± standard deviations (*n* = 3). Values with different superscripts are significantly different vs. control (untreated cells); * *p* < 0.05, ** *p* < 0.01, *** *p* < 0.001, **** *p* < 0.0001 among samples.

To identify the appropriate dose of the hydrogel material with the highest antibacterial property (i.e., wound treatment) but minimal cytotoxicity, it was essential to carry out the biosafety assessment. These tests are absolutely necessary, especially since numerous studies have demonstrated in vitro cytotoxic and cytostatic effects of *C. officinalis* extract in different cell lines [106,107,108,109]. Therefore, the cytocompatibility of unloaded and *C. officinalis*-loaded P/PVA hydrogels was evaluated through in vitro studies on human dermal fibroblasts (HDFa) (exposed for 24 h). MTT assay results (Figure 9) showed that the cytotoxicity of all extract-loaded hydrogels was higher than that observed for empty hydrogels. As expected, a gradual decrease in the HDFa cell viability after incubation with the increase in extract concentrations was noticed. In particular, the cytotoxicity of the samples was directly proportional to the concentration of the *C. officinalis* extract, with the greatest cell viability at the highest dilutions (<200 µg/mL, considering the total elution of *C. officinalis* from the samples after 24 h).

Several studies showed that the increase in the cytotoxicity of *Calendula* extract is related to the cytotoxic effect of some components that become toxic only above a given dose and time exposure [106,110].

## 4. Conclusions

Pullulan/PVA hydrogels containing *Calendula officinalis* extract with antioxidant and antimicrobial properties were designed and then developed for further use as a possible material in wound healing applications. The proposed hydrogel was obtained by a double cross-linking procedure and loaded with *Calendula officinalis* hydroalcoholic extract by a post-loading method. The effect of the plant extract content on the properties of the hydrogel matrix was evaluated.

The results clearly demonstrate the potential of *C. officinalis*-loaded P/PVA hydrogels to be used as a biomaterial for skin damage treatment. First, the hydrogel showed relevant in vitro antioxidant activity against DPPH radicals, and bactericidal and bacteriostatic activity against relevant pathogens. Second, it displayed sustained release properties and no cytotoxicity against HDFa cells was observed.

## Figures and Tables

**Table 1:** Phenolic compounds and triterpenes detected in *Calendula officinalis* extract.

No.	Identified Compound	R_T_ (min)	Column/ Ionization Mode	Major ESI *m*/*z* Observed	Ref.
1	Chlorogenic acid	9.23 24.34	Phenomenex C18 Luna/ESI(−) Mediterranea Sea 18/ESI(+)	707.22 [2M-H]^−^ 353.11 [M-H]^−^ 191.06 [QA-H]^−^ 355.31 [M+H]^+^ 163.14 [M-QA]^+^	[53,54,55]
2	Caffeic acid	11.35	Phenomenex C18 Luna/ESI(−)	179.04 [M-H]^−^ 135.05 [M-CO_2_]^−^	[59]
3	Quercitin-3-O-rhamnosylrutinoside	13.32	Phenomenex C18 Luna/ESI(−)	1511.46 [2M-H]^−^ 755.24 [M-H]^−^	[59,60]
4	Typhaneoside	14.77 42.50	Phenomenex C18 Luna/ESI(−) Mediterranea Sea 18/ESI(+)	769.25 [M-H]^−^ 771.162 [M+H]^+^ 625.35 [M-Rham+H]^+^	[57,58]
5	Quercitin-3-O-rutinoside	15.75 39.68	Phenomenex C18 Luna/ESI(−) Mediterranea Sea 18/ESI(+)	1219.34 [2M-H]^−^ 609.18 [M-H]^−^ 611.34 [M+H]^+^ 465.32 [M-Rham+H]^+^	[59,60]
6	Isorhamnetin 3-O-rutinoside	16.55 43.22	Phenomenex C18 Luna/ESI(−) Mediterranea Sea 18/ESI(+)	1247.37 [2M-H]^−^ 623.20 [M-H]^−^ 625.35 [M+H]^+^ 479.33 [M-Rham+H]^+^	[59,60,62]
7	Quercetin-3-O-galactoside	16.55 45.32	Phenomenex C18 Luna/ESI(−) Mediterranea Sea 18/ESI(+)	927.22 [2M-H]^−^ 463.11 [M-H]^−^ 301.12 [M-Glu-H]^−^ 465.32 [M+H]^+^	[60,61]
8	Quercitin-3-O-rutinoside isomer	17.32	Phenomenex C18 Luna/ESI(−)	1219.34 [2M-H]^−^ 609.17 [M-H]^−^	[59,60]
9	Caffeic acid 3-glucoside	17.58	Mediterranea Sea 18/ESI(+)	343.54 [M+H]^+^ 181.15 [M-Glu+H]^+^	[53,54,55]
10	Isorhamnetin rutinoside	18.01	Phenomenex C18 Luna/ESI(−)	1247.37 [2M-H]^−^ 623.20 [M-H]^−^	[60,62]
11	Dicaffeoylquinic acid	18.43	Phenomenex C18 Luna/ESI(−)	515.14 [M-H]^−^	[61,62]
12	Isorhamnetin-3-O-glucoside	18.97 49.93	Phenomenex C18 Luna/ESI(−) Mediterranea Sea 18/ESI(+)	955.25 [2M-H]^−^ 477.12 [M-H]^−^ 479.32 [M+H]^+^	[56,59,60,62]
13	Hydroxycaffeic acid	34.28	Mediterranea Sea 18/ESI(+)	197.26 [M+H]^+^ 179.13 [M-OH]^+^	[53,54,55]
14	Dicaffeoylquinic acid	18.43	Phenomenex C18 Luna/ESI(−)	515.14 [M-H]^−^ 353.10 [CGA-H]^−^	[61,62]
15	Isorhamnetin 3,7-di-O-β-D-glucopyranoside	46.22	Mediterranea Sea 18/ESI(+)	641.513 M+H]^+^	[53,54,55]
16	Oleanolic acid glucuronide A	26.40	Phenomenex C18 Luna/ESI(−)	1117.57 [M-H]^−^ 793 [M-H-2Hex]^−^	[52,60,63]
17	Oleanolic acid glucuronide A isomer	29.39	Phenomenex C18 Luna/ESI(−)	1117.56 [M-H]^−^ 581.29 [M-H-2Hex-HexUA]^−^	[52,63,64]
18	Oleanolic acid glucuronide C	30.28	Phenomenex C18 Luna/ESI(−)	955.50 [M-H]^−^ 500.26 [M-OA]^−^	[52,63]
19	Oleanolic acid glucuronide D	30.75	Phenomenex C18 Luna/ESI(−)	793.46 [M-H]^−^ 569 [M-H-Hex-H_2_O-CO_2_]^−^	[52]

Notes: [M+H]^+^/[M-H]^−^, value of protonated/deprotonated molecule; Glu—glucose; Hex—hexose (*m*/*z* 162/180); HexUA—hexuronic acid (*m*/*z* 194); OA—oleanolic acid (*m*/*z* 455); Rham—rhamnose (*m*/*z* 146); QA—quinic acid (*m*/*z* 192); CGA—chlorogenic acid (*m*/*z* 354).

**Table 2:** Physico-chemical characteristics of *Calendula officinalis* extract (hydroalcoholic 1:5 solution).

TPC (mg GAE/g)	TFC (mg QE/g)	TTPC (mg OAE/g)	DPPH, IC_50_ (μg/mL)		
			*C. officinalis* Extract	Ascorbic Acid	Quercetin
15.03 ± 0.45	79.42 ± 2.92	53.58 ± 0.47	122.67 ± 4.5	3.1 ± 0.12	2.3 ± 0.14

TPC—total phenolic content expressed as mg gallic acid equivalents (GAE)/g dry plant extract; TFC—total flavonoid content expressed as mg quercetin equivalents (QE)/g dry plant extract; TTPC—total triterpenes content expressed as mg oleanolic acid equivalents (OAE)/g dry plant extract. Results are expressed as mean ± standard deviation of three (*n* = 3) determinations.

**Table 3:** Composition of the initial mixture and P/PVA hydrogels.

Sample Code	Initial Mixture			Final Composition			
	P/PVA Ratio (%, *w*/*w*)	STMP/(P + PVA) Ratio (*w*/*w*)	NaOH/(P + PVA) Ratio (*w*/*w*)	GF * (%, *w*/*w*)	P (%, *w*/*w*)	PVA (%, *w*/*w*)	PO_4_^2− ^ Content ** (meq./g)
H #0	25/75	0.125/1	0.1/1	65.44 ± 3.9	36.08 ± 2.45	63.92 ± 2.45	0.74 ± 0.06

* GF represents the gel fraction = W_2_/W_1_, where W_2_ and W_1_ are the weight of the sample after and before extraction with water for 3 days, respectively. ** determined by acid–base conductometric titration. Results are expressed as mean ± standard deviation of three (*n* = 3) determinations.

**Table 4:** Loading parameters of hydrogels with *Calendula officinalis* extract.

Sample Code	*C. officinalis* Conc. (%, *w*/*v*)	Gravimetric Ratio Extract/H (*w*/*w*)	LC (mg *C. officinalis*/g H)	TPC (mg GAE/g H)	TTPC (mg OAE/g H)
H #1	5	1/1	211.4 ± 8.9	10.02 ± 0.73	9.98 ± 0.78
H #2	10		334.4 ± 4.6	14.36 ± 1.09	12.18 ± 1.07
H #3	20		736.7 ± 2.1	24.33 ± 1.04	22.87 ± 1.11

H—hydrogel; LC—loading capacity determined by Equation (5); TPC—total phenolic content expressed as mg gallic acid equivalents (GAE)/g hydrogel; TTPC—total triterpenes content expressed as mg oleanolic acid equivalents (OAE)/g hydrogel. Results are expressed as mean ± standard deviation of three (*n* = 3) determinations.

**Table 5:** Mechanical properties of unloaded and *Calendula officinalis*-loaded P/PVA hydrogels.

Sample Code	Young’s Modulus (KPa)	Compressive Strength (KPa)	Maximal Adhesive Force F_max_ (N)
H #0	42.53 ± 5.22	61.67 ± 6.47	0.37 ± 0.04
H #1	78.87 ± 3.40 ^b^	81.75 ± 3.44 ^a^	0.38 ± 0.04
H #2	69.06 ± 1.13 ^b^	76.25 ± 2.97	0.43 ± 0.02 ^a^
H #3	47.56 ± 4.80	66.33 ± 3.60	0.56 ± 0.03

Results are expressed as means ± standard deviation (S.D.) of three (*n* = 3) independent experiments. Values with different superscripts are significantly different vs. control (H #0), *p* < 0.05.

**Table 6:** Antibacterial and antifungal activities of *Calendula officinalis*-loaded hydrogels.

Mean Value and Standard Deviation of the Inhibition Zones (mm) *					
Species	C (+)	C (−)	H #1	H #2	H #3
		*C. officinalis* Extract Content (mg)			
		0	2.1	3.9	10.5
*S. aureus* ATCC25923	22	5	11 ± 0.3	12 ± 0.25	13 ± 0.35
*Escherichia coli* ATCC 25922	24	5	11.5 ± 0.1	11.6 ± 0.4	12 ± 0.5
*P. aeruginosa* 9027 ATCC	22	5	5 ± 0.1	5 ± 0.2	15 ± 0.1
*C. albicans* ATCC 90028	16	5	6 ± 0.25	7 ± 0.3	8 ± 0.4

* Inhibition zones including the disc diameter of 5 mm; resistant: d ≤ 12 mm, intermediate: d = 13–14 mm, susceptible: d ≥ 15 mm [101]. C(+) = Gentamicin standard disc (10 µg), C(−) = unloaded P/PVA hydrogel (H #0). Data represent the mean of three (*n* = 3) independent experiments.

**Table 7:** Reduction population (log CFU) of *S. aureus*, *E. coli*, *P. aeruginosa,* and *C. albicans* after treatment with *Calendula officinalis*-loaded hydrogels.

Time (h)	Sample		Log Reduction/% Reduction			
		Species	*S. aureus*	*E. coli*	*P. aeruginosa*	*C. albicans*
24	H #1		4.87/100	4.87/100	2.96/99.89	3.92/99.98
	H #2		4.87/100	4.87/100	3.20/99.94	4.87/100
	H #3		4.87/100	4.87/100	4.87/100	4.87/100
72	H #1		4.87/100	2.17/99.33	2.39/99.59	2.95/99.88
	H #2		4.57/99.99	2.37/99.58	2.53/99.70	2.98/99.89
	H #3		4.57/99.99	4.57/99.99	4.57/99.99	4.87/100

*Calendula officinalis* extract content: H #1 = 2.1 mg; H #2 = 3.9 mg, and H #3 = 10.5 mg. Data represent the mean of three (*n* = 3) independent experiments.

## Data Availability

Data are available on request.

## Supplementary Materials

The following supporting information can be downloaded at https://www.mdpi.com/article/10.3390/pharmaceutics15061674/s1, Figure S1. HPLC-DAD chromatogram (wavelengths of 280 nm) for standard mixture containing 16 phenolic compounds (gallic acid, 3,4 DHB, chlorogenic acid, vanillic acid, caffeic acid, syringic acid, p-coumaric acid, cinnamic acid, cynarin, quercetin 7-rhamnoside, luteolin 7-o-glucoside, isoquercetin, apigenin 7-glucoside, kaempferol 7-O-glucoside, fisetin, and quercetin); Figure S2. ESI(+) MS extracted ion chromatograms (EIC) and relevant mass spectra for some detected phenolic compounds in *Calendula officinalis* extract; Figure S3. Representative ESI(-) mass spectrum for identified triterpenes: oleanolic acid glucuronide D (*m*/*z* 793), oleanolic acid glucuronide C (*m*/*z* 955), and oleanolic acid glucuronide A (*m*/*z* 1117). Loss of hexose (162 Da), hexuronic acid (193 Da), and oleanolic acid (455 Da); Figure S4. DPPH radical scavenging and estimation of IC_50_ value of *Calendula officinalis* extract, quercetin, and ascorbic acid; Figure S5: Antibacterial activity of unloaded and *Calendula officinalis*-loaded P/PVA hydrogels against *S. aureus*, *E. coli*, *P. aeruginosa*, and *C. albicans* through well-diffusion assay.
