# Supplementary material for: Pullulan/Poly(vinyl alcohol) Hydrogels Loaded with Calendula officinalis Extract: Design and In Vitro Evaluation for Wound Healing Applications
Source: Pharmaceutics. 2023 Jun 7;15(6):1674. doi: 10.3390/pharmaceutics15061674 (PMC10301438; doi:10.3390/pharmaceutics15061674)
Supplement: Supplementary file 1 [file pharmaceutics-15-01674-s001.zip › pharmaceutics-2367489-supplementary.pdf]

Electronic Supplementary Information (ESI)

Pullulan/poly(vinyl alcohol) hydrogels loaded with *Calendula officinalis* extract: design and in vitro evaluation for wound healing applications

Irina Mihaela Pelin [1], Mihaela Silion [1], Irina Popescu [1], Cristina M. Rîmbu [2], Gheorghe Fundueanu [1] and Marieta Constantin [1,*]

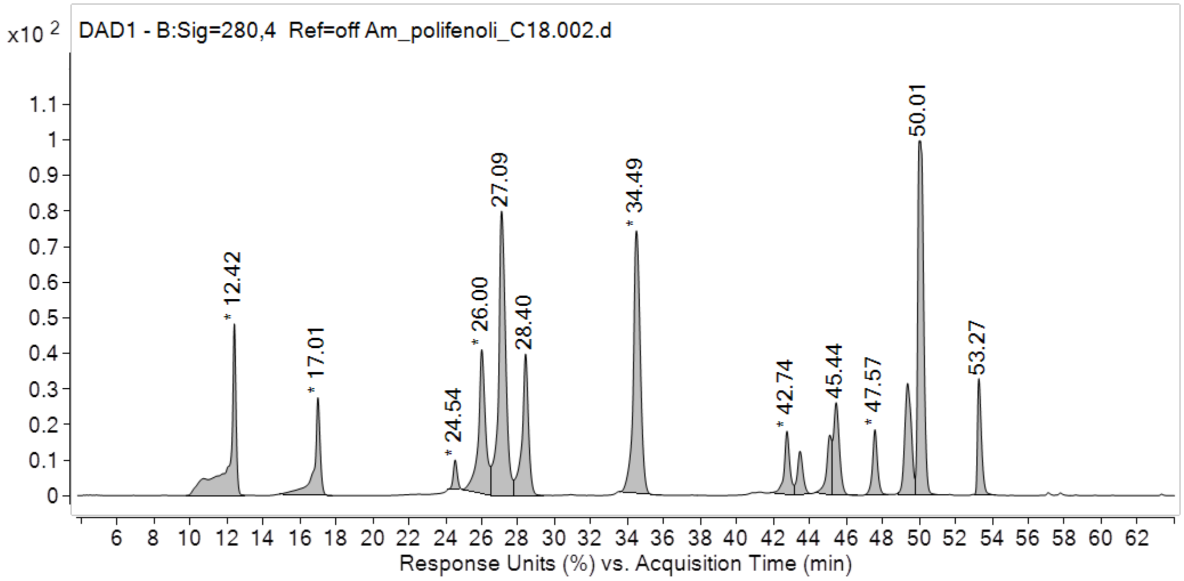

Figure S1. HPLC-DAD chromatogram (wavelengths of 280 nm) for standard mixture containing 16 phenolic compounds (gallic acid, 3,4 DHB, chlorogenic acid, vanillic acid, caffeic acid, syringic acid, p-coumaric acid, cinnamic acid, cynarin, quercetin 7-rhamnoside, luteolin 7-o-glucoside, isoquercetin, apigenin 7-glucoside, kaempferol 7-O-glucoside, fisetin, quercetin).

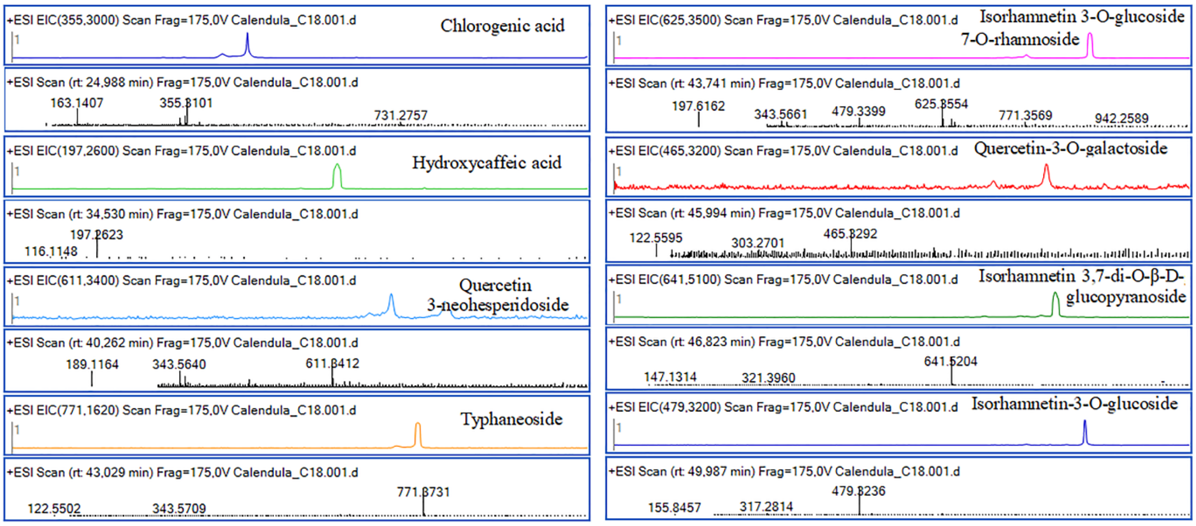

Figure S2. ESI(+) MS extracted ion chromatograms (EIC) and relevant mass spectra for some detected phenolic compounds in *Calendula officinalis* extract.

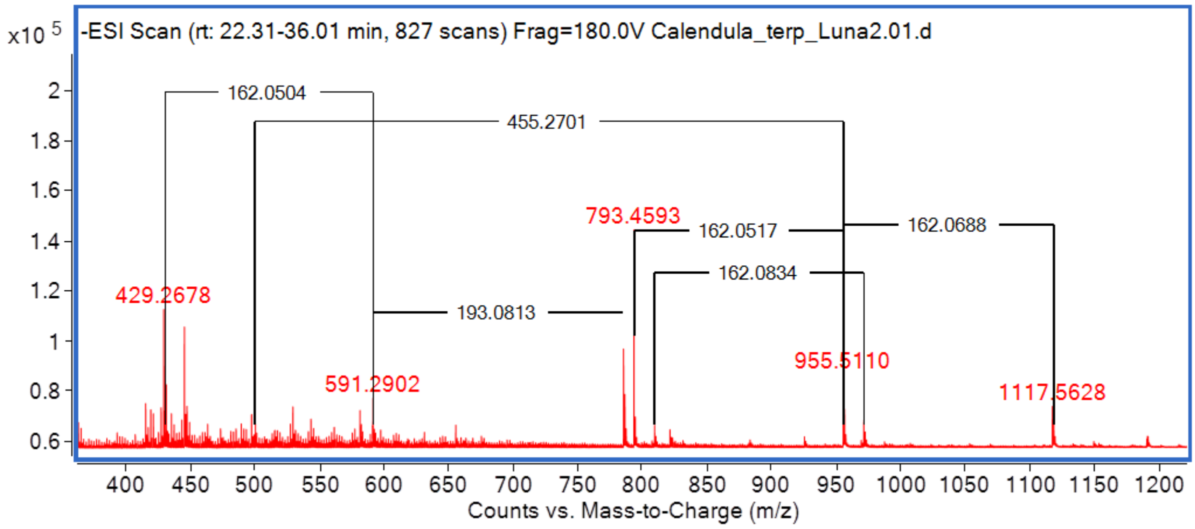

Figure S3. Representative ESI(-) mass spectrum for identified triterpenes: oleanolic acid glucuronide D (m/z 793), oleanolic acid glucuronide C (m/z 955), and oleanolic acid glucuronide A (m/z 1117). Loss of: hexose (162 Da), hexuronic acid (193 Da), oleanolic acid (455Da).

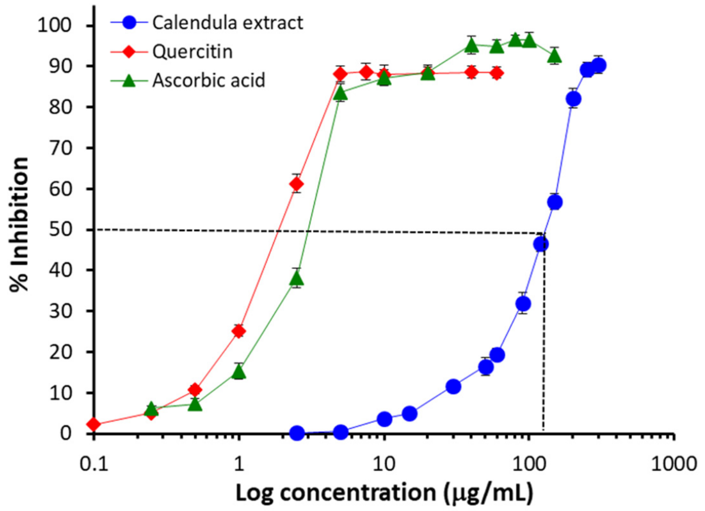

Figure S4. DPPH radical scavenging and estimation of IC_{50} value of *Calendula officinalis* extract, quercetin and ascorbic acid.

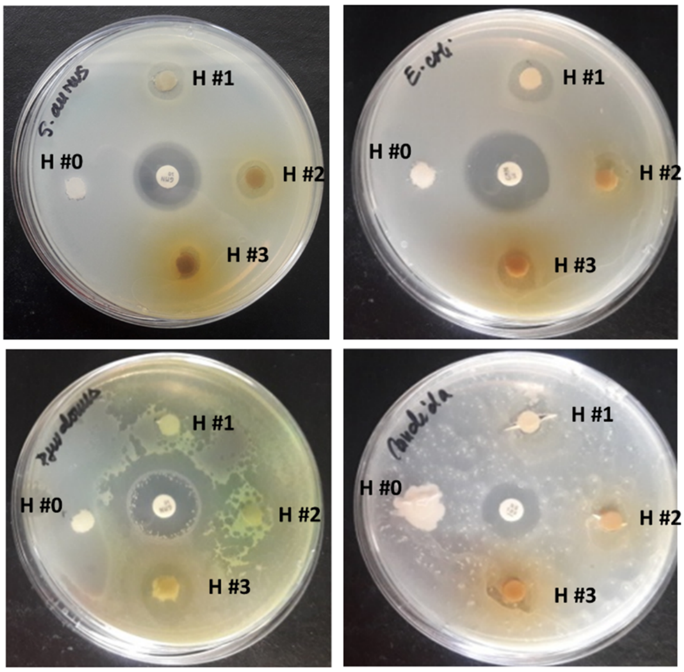

Figure S5. Antibacterial activity of unloaded and *Calendula officinalis*-loaded P/PVA hydrogels against *S. aureus*, *E. coli*, *P. aeruginosa* and *C. albicans* through well diffusion assay.
